# ImmuneDB, a Novel Tool for the Analysis, Storage, and Dissemination of Immune Repertoire Sequencing Data

**DOI:** 10.3389/fimmu.2018.02107

**Published:** 2018-09-21

**Authors:** Aaron M. Rosenfeld, Wenzhao Meng, Eline T. Luning Prak, Uri Hershberg

**Affiliations:** ^1^School of Biomedical Engineering Science and Health Systems, Drexel University, Philadelphia, PA, United States; ^2^Department of Pathology and Laboratory Medicine, Perelman School of Medicine, University of Pennsylvania, Philadelphia, PA, United States; ^3^Department of Microbiology and Immunology, College of Medicine, Drexel University, Philadelphia, PA, United States; ^4^Department of Human Biology, Faculty of Sciences, University of Haifa, Haifa, Israel

**Keywords:** next-generation sequencing, antibody repertoire analysis, bioinformatics, B-cell receptor, database

## Abstract

ImmuneDB is a system for storing and analyzing high-throughput immune receptor sequencing data. Unlike most existing tools, which utilize flat-files, ImmuneDB stores data in a well-structured MySQL database, enabling efficient data queries. It can take raw sequencing data as input and annotate receptor gene usage, infer clonotypes, aggregate results, and run common downstream analyses such as calculating selection pressure and constructing clonal lineages. Alternatively, pre-annotated data can be imported and analyzed data can be exported in a variety of common Adaptive Immune Receptor Repertoire (AIRR) file formats. To validate ImmuneDB, we compare its results to those of another pipeline, MiXCR. We show that the biological conclusions drawn would be similar with either tool, while ImmuneDB provides the additional benefits of integrating other common tools and storing data in a database. ImmuneDB is freely available on GitHub at https://github.com/arosenfeld/immunedb, on PyPi at https://pypi.org/project/ImmuneDB, and a Docker container is provided at https://hub.docker.com/r/arosenfeld/immunedb. Full documentation is available at http://immunedb.com.

## Introduction

The study of immune cell populations has been revolutionized by next-generation sequencing. It is now commonplace to have hundreds of thousands or even millions of sequences from a single sample or individual (1, 2). With this increase in experimental data output, many tools have been created for pre-processing sequences (3), germline association and clonal inference (4–7), and post-processing analysis (8, 9). Lacking from this space, however, is a system to store fully-annotated sequences, their inferred germline sequences, clonal associations, and study-specific metadata. This paper describes ImmuneDB (10) and introduces new features added since its original publication including: additional importing & exporting formats, a more flexible metadata system, extra clonal assignment methods, integration of a novel allele detection tool (11), and the ability to analyze other species and light chains. ImmuneDB provides an easy to use immune-receptor sequence database, which has been optimized for and tested with datasets of up to hundreds of millions of sequences (1). It can take as input raw FASTA/FASTQ sequence files, or import pre-annotated sequences from an array of formats including the Change-O data standard (5) and the AIRR data standard currently being implemented and further refined (2). With either method, it can infer clonal associations, calculate selection pressure, generate lineages, and make all resulting information available both from the command line and as a web-interface. For interoperability with other systems, ImmuneDB can output data in AIRR, Change-O, VDJtools, and genbank formats. ImmuneDB's usage of MySQL also allows for rapid querying and data-sharing using a variety of existing tools.

## Materials and methods

**Figure 1:**
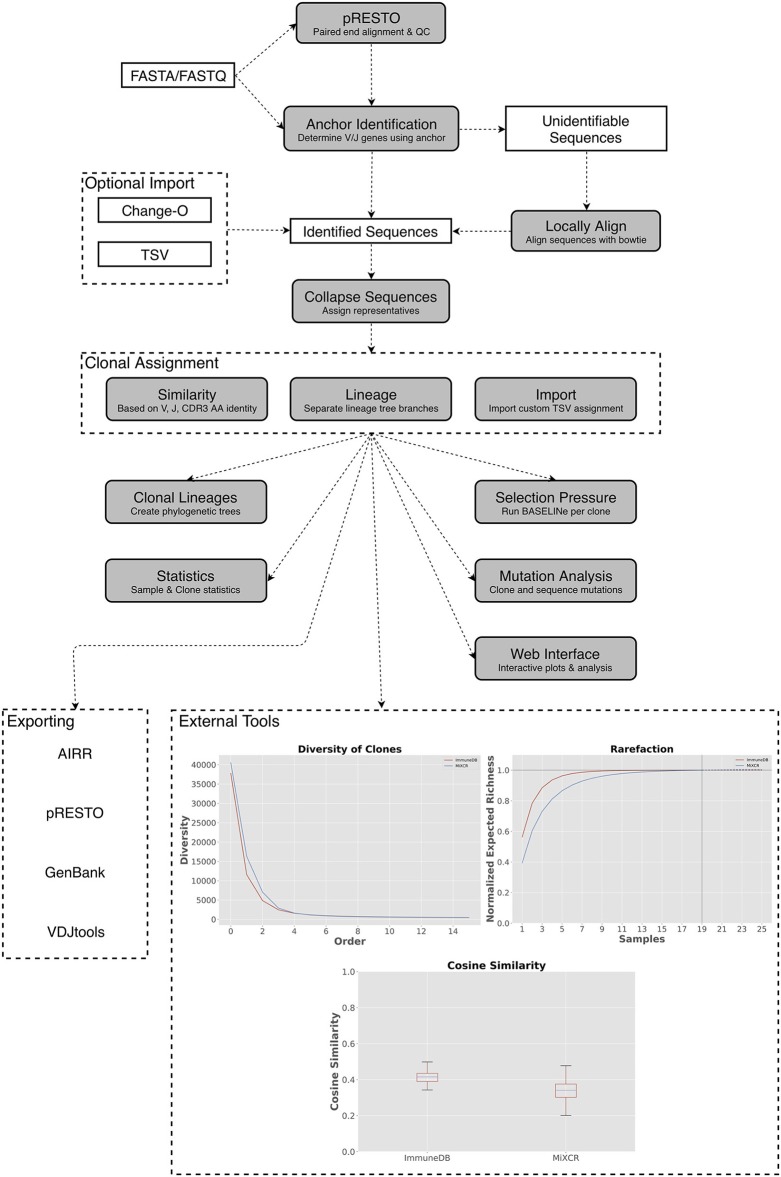
A general overview of the ImmuneDB pipeline. To start, sequences are optionally pre-processed with pRESTO to remove poor-quality sequences and mask bases below a user-defined threshold. Next, using a conserved region anchoring method, sequences are either assigned V- and J-genes or labeled as “unidentifiable” which optionally can be corrected by local alignment. After gene assignment, sequences are collapsed across samples and grouped into clones based on one of three methods (see text). Lastly, downstream analyses such as selection pressure, and lineage construction are performed. A web interface is available to browse the resulting data and analyzed data can be exported in a variety of formats. Inset: Examples of downstream analysis: cosine similarity between inferred B-cell rearrangements in tissue samples from an organ donor, diversity (calculated as defined in Equation 1) plotted at different orders from the same tissue samples; rarefaction calculated for B-cell rearrangements amplified from colon samples.

The methods below describe the ImmuneDB pipeline in the context of human B-cell heavy chain rearrangements. We then extend the methods to T cells, light chains, and other species (Figure 1).

### Computer hardware and software requirements

ImmuneDB is primarily written in Python and can therefore run on most common Unix-based operating systems (including macOS). Local installation of the version described in this paper (v0.24.1) requires Python 3.5+, although legacy versions support Python 2.7. The setup will automatically install all Python library dependencies. Additionally, MySQL (or a drop-in replacement like MariaDB) is required, although it need not run on the same host as ImmuneDB.

Optional steps require installation of additional external tools. Local alignment requires Bowtie 2 (12), lineage construction depends on Clearcut (13), selection pressure calculations utilize BASELINe (9), novel gene detection requires TIgGER (11), and the web-frontend exists in a separate repository^1^.

Alternatively, a Docker image^2^ is available with all these dependencies pre-installed along with helper scripts, and is therefore the recommended method for using ImmuneDB.

Hardware requirements depend on the input data, but as a general guideline it is recommended that ImmuneDB be run on a machine with enough available memory to store at least three times the largest input sample (e.g., for a 5 Gb input file, 15 Gb of memory should be available). Any number of cores are acceptable and ImmuneDB uses Python's multiprocessing library to utilize as many cores as possible.

### Germline reference database

ImmuneDB can use any IMGT aligned V- and J-gene database which it accepts as a pair of FASTA files. We suggest always using the most recent IMGT/GENE-DB (14) database including only functional germlines.

### License

ImmuneDB is released under the GNU General Public License, version 3^3^ allowing for unlimited use, modification, and distribution under the same license and with any changes explicitly stated.

### The immuneDB pipeline

ImmuneDB is comprised of sequential steps, run via the command line, that generate a database with analyzed immune receptor data as shown in Figure 1. Before running ImmuneDB, it is recommended that pRESTO (3) be used for quality control and, when applicable, paired-read assembly. ImmuneDB itself begins with V- and J-gene identification and optional local-alignment. Then, duplicate sequences are identified across samples originating from the same subject. These sequences are then grouped into clones using one of three methods of clonal inference (described in section Clonal Inference). Finally, aggregate statistics are generated and results can be exported, explored in a web browser, or further analyzed with an integrated set of downstream-analysis tools.

Each step of the pipeline is detailed in this section along with an example of the command to run. In all cases passing the –help flag will list all possible parameters and their default values (if any).

#### Raw data processing

Before running the ImmuneDB pipeline itself, raw FASTQ reads from a sequencer should be quality controlled using pRESTO. First, sequences are trimmed of poor-quality bases on the end farthest from the primer where base call confidence tends to degrade. Using default parameters, sequences are then trimmed to the point where a window of 10 nucleotides has an average quality score of at least 20. If reads are paired, the next step is to align the R1 and R2 reads into full-length, contiguous sequences. Short sequences, those with less than 100 bases, are then removed from further analysis. Finally, any base with a quality score less than 20 is replaced with an *N* and any sequence containing more than 10 such bases is removed from further analysis. In the case of FASTA input which has no quality information, only paired-end assembly and short sequence removal are recommended. A detailed script for running this process can be found in Rosenfeld et al. (15).

After this process, the remaining filtered sequences are presumed to be of adequate quality for germline inference and clonal assignment.

#### Creating a database

ImmuneDB allows users to separate their datasets into individual ImmuneDB *project*, each with their own database. To create a properly structured MySQL database, the immunedb_admin command is used:

$ immunedb_admin create db_name ~/configs

Running this command with db_name replaced with an appropriate name will create a database named db_name and create a configuration file in ~/configs with information for the remainder of the pipeline to access it. Specifically, it records a unique username and password for the database so each project you create is separated from others. Database names must consist of only alphanumeric characters, integers, and underscores.

#### Sample metadata assignment

Each ImmuneDB project is designed to house data across many samples and subjects. It is recommended that each quality-controlled FASTA/FASTQ file contains the sequences from one biologically independent sample. This implies that, if a given sequence is found in multiple independent samples, it actually occurred in multiple cells. Although not recommend, ImmuneDB will still operate normally if samples originated from multiple sequencing runs of the same PCR aliquot. However, many measures of sequence abundance and clone size break down under this conditions [see section Sequence Collapsing (copies, uniques, instances) for discussion].

For the ImmuneDB pipeline, some metadata about each sample are required: a unique sample name and a subject identifier. Samples with the same subject identifier came from the same source organism. Additional custom metadata (e.g., cell subset, tissue) can be attached to each sample, which can be useful for later analysis and grouping.

To generate a template metadata file in the directory with the FASTA/FASTQ files for processing, the user runs:

$ immunedb_metadata –use-filenames

This will generate a metadata.tsv file that should be further edited with the appropriate information, and will be used in the next step of the pipeline. The optional -use-filenames flag pre-populates the sample names with the associated filename, stripped of its.fasta or.fastq extension.

#### Germline assignment (anchoring, local alignment)

The first portion of the ImmuneDB pipeline infers V- and J-genes for each set (sample) of quality-filtered reads using the approach in Zhang et al. (4). This method was chosen because it is quicker than local-alignment and works for the majority of sequences which are not mutated in conserved regions flanking the CDR3. Given a small number of restrictions detailed in the documentation, this method can accept user-defined germlines so long as they are properly IMGT numbered (16). Specifics about the numbering scheme can be found at^4^.

For each sequence, the anchor method first searches for a conserved region of the J gene. If it is found, all germline J-gene sequences are compared to the same region in the sequence, and the one with the smallest Hamming distance (17) is assigned as the putative J gene. Since ImmuneDB requires sequences to have a J- and V-gene assignment to be included in clones, if no anchor is found the sequence is marked as unidentifiable and is excluded from V-gene assignment for efficiency.

Then, a conserved region near the 3′ end of the V-segment is used to position each sequence correctly relative to the IMGT numbered germline sequences. As with J-genes, each germline sequence is then compared using Hamming distance, and the one with the smallest distance is assigned as the putative V gene. If the conserved region is not found, the sequence is marked as unidentifiable and excluded from the rest of the anchoring process.

After every sequence is assigned a V and J gene (or marked as unidentifiable) the average mutation frequency and sequence length are calculated. For each sequence, other germline genes which are statistically indistinguishable from the putative genes are added as “gene-ties.” Thus, each sequence may have multiple V- and J-gene assignments.

As a post-identification quality control step, ImmuneDB then marks sequences with a low V-germline identity (defaulting to 60%) as unidentifiable. Further, any sequence which has a window of 30 nucleotides with less than 60% germline identity is marked as a “potential insertion or deletion”.

To run this step, the user enters the following commands:


$ immunedb_identify /path/to/config.json \
       /path/to/v_germlines.fasta \
       /path/to/j_germlines.fasta .

After this command finishes, the anchoring portion of alignment is complete. Due to insertions or deletions, mutations in the conserved regions, and other anomalies, there are generally sequences which cannot be identified with this approach. To rectify such sequences, ImmuneDB can then optionally use Bowtie 2 (12) to attempt local-alignment on each of these sequences. Any insertion or deletions that Bowtie 2 finds are also stored with the sequence. The command to locally align sequences is similar to identification:


$ immunedb_local_align /path/to/config.json \
       /path/to/v_germlines.fasta \
       /path/to/j_germlines.fasta .


#### Sequence collapsing (copies, uniques, instances)

After sequences are assigned V and J genes, sequences that differ only at N positions—those which had low quality calls from the sequencer—are collapsed within each sample resulting in one set of unique sequences per sample. Each unique sequence maintains a count called “copy number” of how many duplicates occurred in the sample. Then, all the sample-level unique sequences within the same subject are compared to one another and duplicates are marked and collapsing information is stored.

After this process, each subject-level unique sequence has two fields associated with it: *total copies* and *instances*. When samples are biologically distinct, which is recommended in section Sample Metadata Assignment, the instance count of a sequence is the number of samples in which that sequence occurred (which can be interpreted as the lower bound on number of cells that contained that sequence) and the total copies is the number of duplicates across all samples. Although the latter is subject to PCR artifacts, it can give an indication of true sequence abundance. Alternatively, when samples are *not* biologically independent, the instances of a sequence no longer give a bound on cell count and the copy number of a sequence may be inflated, leading to skewed sequence and clone abundance calculations.

An overview of the terms copy number, instances, and unique sequences is provided in Table 1.

**Table 1:** Summary of terms for sequence collapsing.

**Term**	**Description**
Unique Sequence	A distinct sequence across an entire subject
Instance	A distinct sequence within a single sample
Copy number	The number of raw reads that are associated with an instance or unique sequence

*A description of the three terms used to indicate sequence or clonal sizes*.

To run the collapsing process, run:

$ immunedb_collapse /path/to/config.json

#### Novel allele detection and correction

The ImmuneDB gene identification process assumes the germline allele database provided, from IMGT or another repository, are indeed those present within the subjects being analyzed. Users can add or remove genes as needed by modifying the germline FASTA files input into ImmuneDB. However, in many cases it may not be known *a priori* which genes are or if the subjects have novel germline alleles. To determine which genes are present in a dataset, ImmuneDB may optionally run TIgGER (11) on sequences to identify potential differences from the standard germline database. To do so, the identification and collapsing processes above is run with a presumed germline database followed by:


$ immunedb_export /path/to/config.json
       changeo \
      ––min-subject-copies 2
$ immunedb_genotype /path/to/config.json \
       /path/to/v_germlines.fasta


This exports the sequences, as identified with the presumed germline genes, with at least two copies in the subject and then runs TIgGER. If novel alleles are found, a new set of input germlines is generated, and ImmuneDB can be re-run with these germline reference genes.

#### Clonal inference

ImmuneDB incorporates two methods of clonal inference, all of which start with the same set of sequences: the subject-level unique sequences calculated previously. By default, only such sequences with a copy number of at least two are considered eligible for clonal assignment. This eliminates some of the sequences that potentially arose from sequencing error and could cause spurious construction of clones. After this process, each clone has three defined levels of size. The number of u*nique sequences* are the number of distinct sequences that comprise a clone. *Copies* and *instances* are defined as the sum of *copies* and *instances* over the clone's constituent unique sequences. These clone size metrics are reviewed in detail in Rosenfeld et al. (15).

#### CDR3 similarity

The first method of clonal inference is for B cells. It uses CDR3 similarity to group sequences from the same subject with the same gene assignments and CDR3 length into clones. Initially an empty list of clones *C* is created. Let *S* be the set of all subject-level unique sequences.

Each sequence *s*∈*S* is visited in order of decreasing copy number. If there is a clone *c*∈*C* such that every sequence already assigned to *c* has the same V gene, J gene, CDR3 length in nucleotides, and has 85% CDR3 amino-acid similarity, *s* is added to the clone *c*. Otherwise, a new clone is added to *C* containing only *s*. This results in a set of clones such that all the sequences in a clone share the same gene assignments, CDR3 length, and pairwise are at least 85% similar in the CDR3. The percent similarity can be tweaked by the user as necessary.

This method of clonal inference can be run with:


$ immunedb_clones /path/to/config.json \
        similarity


#### Lineage separation

The newest method of clonal assignment in ImmuneDB is based on (18). For each subject, sequences are placed into buckets based on their V gene, J gene, and CDR3 length in nucleotides. Then, a lineage is made out of each bucket. Working from the root node of the lineage (the germline) each edge is traversed until a specified number (by default four) mutations accumulates. The subtree starting at that point is then grouped into a clone. This method, unlike similarity-based methods, is order-agnostic and can be run with:

$ immunedb_clones /path/to/config.json \
        lineage


#### Selection pressure

After clonal inference, ImmuneDB can optionally use BASELINe (9) to estimate clonal selection pressure. It first runs on each clone as a whole, providing an overview of selection pressure in the framework and complementary regions. Then, it runs independently on the subset of sequences that occur in each sample. This can be useful when a clone spans multiple samples with various biological features. For example, a clone may appear in samples from different tissues or cell subsets.

To run BASELINe via ImmuneDB, the path to the Baseline_Main.r script must be specified.


$ immunedb_clone_pressure /path/to/config.json \
       /path/to/Baseline_Main.r


#### Lineages

**Figure 2:**
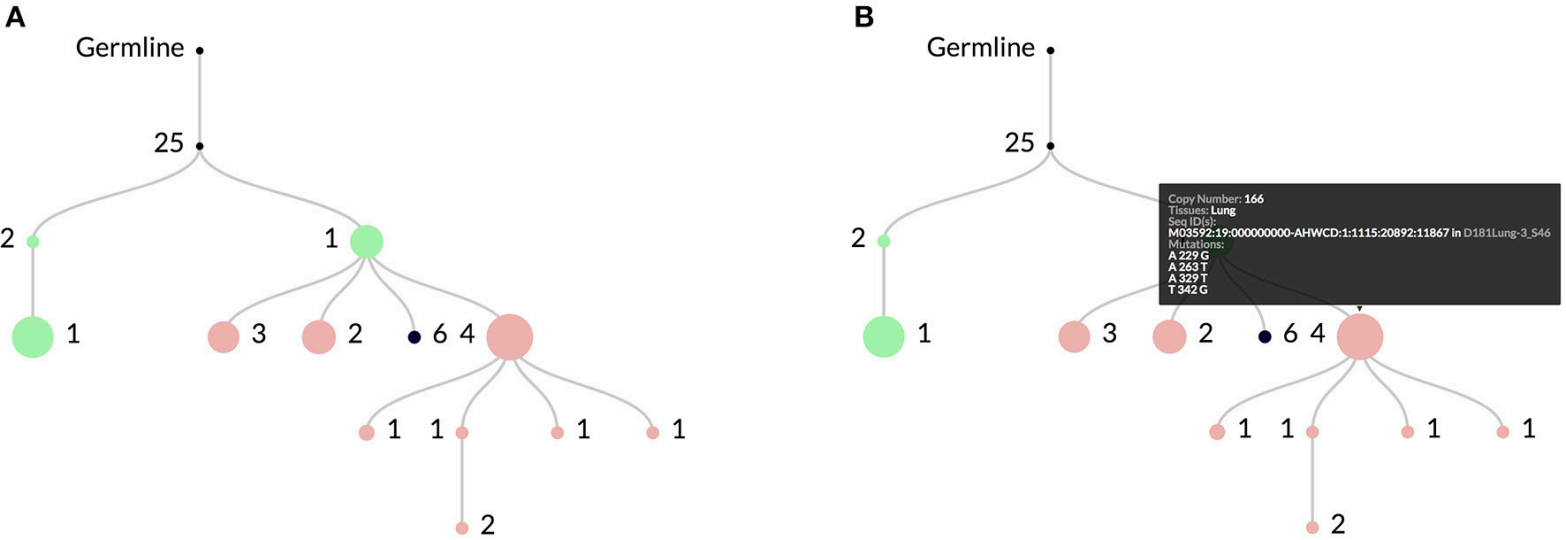
In **(A)** an example clonal lineage as viewed through the web interface for ImmuneDB. Node diameter is proportional to the total number of sequence copies at that node and the edge numbers show the number of mutations between the parent and child nodes. The colors indicate the tissue(s) that make up the sequences at the node. In this case green is Bone Marrow, red is Lung, and black is a combination of both. As shown in **(B)**, hovering over a node gives more information such as the specific mutations, copy number, and sequence metadata.

ImmuneDB integrates Clearcut (13) to infer clonal lineages using neighbor-joining. For each clone, a lineage is constructed and every node maintains information about its associated sequence, as shown via the web-interface in Figure 2. This process can be parameterized in different ways including filtering sequences or mutations that occur less than a set number of times. Generally, it is recommended to run Clearcut excluding mutations that happen exactly once with:

$ immunedb_clone_trees
       /path/to/config.json \
       /path/to/clearcut \
       ––min-count 2


**Figure 3:**
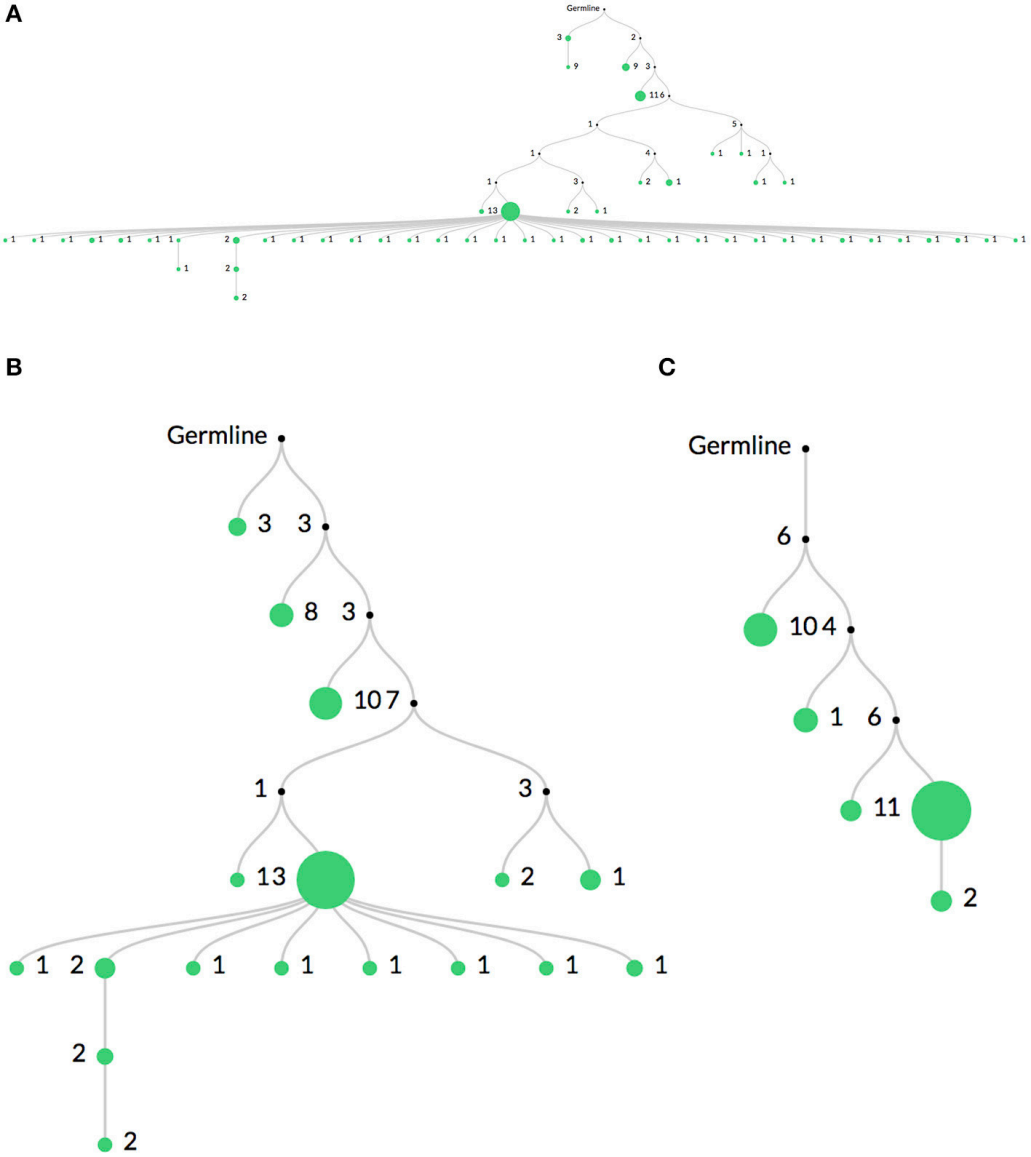
Comparison of mutation thresholding on lineage shape. A comparison of a single clonal lineage constructed from bulk IgH V-region sequencing data where **(A)** all mutations are included, **(B)** only mutations found in at least 2 sequences are included, and **(C)** only mutations in at least 5 sequences are included. The drastic elimination of leaf nodes between **(A)** and **(B)** indicate that they are most likely due to sequencing error. In very deeply sequenced samples or in malignancies where clones are very large, an even higher threshold may be required, such as in **(C)**, since the same sequencing error may occur multiple times.

Figure 3 shows the same clone's lineage constructed with Figure 3A no mutation threshold, Figure 3B a threshold requiring mutations to occur in at least 2 sequences, and Figure 3C a threshold requiring mutations to occur in at least 5 sequences. The large expansion of nodes in Figure 3A is likely due to sequencing error. A higher threshold like in Figure 3C may be useful when there is high sequencing depth or when clones are extremely large (such as in some hematopoietic malignancies). In these cases it is quite likely that the same sequencing error will occur multiple times. However, thresholding mutations means the lineages may not accurately reflect recent or rare clonal events.

#### Web interface

ImmuneDB comes with a web interface for browsing analyzed data. It allows users to group and filter data to generate interactive plots, view clones, and inspect sequences. It is primarily intended to explore data at a high-level, visualizing individual samples or comparing different samples in various ways. The command line tools can then be used for more fine-grained analysis. An example interface can be found at http://immunedb.com/tissue-atlas.

To utilize the web interface using the Docker container simply run the following and open http://localhost:8080 in a browser:

$ serve_immunedb.sh /path/to/config.json

Information about running the web interface without the Docker container or with more sophisticated configurations, such as hosting multiple databases, are described in the documentation.

### Importing gene assignments and clonal inference from other AIRR tools

Although ImmuneDB has the features to fully analyze sequences from raw reads through clonal assignment, a concerted effort has been made to allow users to import both identified sequences and clonal assignments from other tools. For pre-identified sequences, ImmuneDB can import files in the Change-O data format (5) with:

$ immunedb_import /path/to/config.json \
       /path/to/v_germlines.fasta \
       /path/to/j_germlines.fasta \
       /path/to/changeo_files


Note that this requires a metadata file identical to that needed by the identification step.

Clonal assignments can be imported from either ImmuneDB-identified sequences or imported sequences. First, the command below is run to output a template file with a list of clonal-assignment eligible sequences:


$ immunedb_clone_import /path/to/config.json \
       ––action export sequences.tsv


Users then fill in the clone_id column in sequences.tsv as they desire and import it back into ImmuneDB with:


$ immunedb_clone_import /path/to/config.json \
       ––action import sequences.tsv


Assuming that no constraints are broken (clones must still have the same V gene and J gene and originate from the same subject), the custom clonal assignment will then be accepted by ImmuneDB.

As members of the AIRR Community (19), the authors will continue to integrate data standards (2) as they are defined.

### Aggregate analysis and data export

ImmuneDB automatically aggregates data for some common analyses in the last step of the pipeline with:

$ immunedb_clone_stats /path/to/config.json

$ immunedb_sample_stats /path/to/config.json

This auto-generated, aggregate analysis is not exhaustive, and is meant to provide sufficient data for the web-interface and to guide further investigation. To assist with this, ImmuneDB allows users to easily export all portions of the analyzed dataset in useful, common formats. Specifically, ImmuneDB has integrated export capabilities for the Change-O (5),vdjtools (8), genbank, and FASTA/FASTQ formats. This enables users to quickly use common downstream analysis tools including VDJtools and those included with the Immcantation Framework^5^, or submit their datasets in the AIRR-compliant GenBank format. The basic template for this command is as follows, replacing the term **format** with changeo, vdjtools, or genbank:


$ immunedb_export /path/to/config.json \
       **format**


### Applications to other data types

#### T-cells

ImmuneDB can analyze T-cell receptor sequences, in addition to B-cell receptor sequences. When compared to B-cell analysis, the two changes necessary in the pipeline for T-cell analysis are to use T-cell germline sequences during germline assignment and to specify the T-cell method during clonal inference. The T-cell method groups sequences with the same V gene, J gene, and 100% CDR3 nucleotide identity into clones. Like the B-cell similarity method described in section CDR3 Similarity, the T-cell method does not take into account any mutations in the V- and J genes. In the case of T cells, mutations are assumed to be experimental artifacts as T cell receptors do not undergo somatic hypermutation due to lack of activation-induced cytidine deaminase (AID). Putative T-cell clones may be comprised of sequences which appear to differ in the V- and J-gene sequences. Spurious intra-clonal diversification is likely from sequencing error whereas consistent divergence from the germline within a clone likely arises from allelic differences from the germline database. The latter case can be corrected with TIgGER as described in section Novel Allele Detection and Correction.

#### Light chains

Because ImmuneDB does not attempt to determine D-genes for sequences during germline assignment, light-chains are naturally supported when a proper germline database of the V- and J genes are provided. At the present, the germline genes for kappa and lambda chains must be placed in separate files and run independently. This restriction will be lifted in future versions. Additionally, because of lower junctional diversity, it is recommended that clonal assignment be considered. For example, when using the similarity method, it is likely appropriate to lower the default amino-acid similarity threshold to a value below 85%.

#### Other species

Species other than humans are supported by ImmuneDB, but with two restrictions. First, for the built-in anchoring method for gene identification, germline genes must have conserved anchoring points as described in Zhang et al. (4) and be IMGT aligned. Second, the length of all J genes past the 3′ end of the CDR3 must be fixed, which is the case for all species currently in the IMGT database.

## Comparison to MiXCR

It is difficult to verify the results of clonal and germline association methods as there is no agreed upon gold standard. We attempt to associate different types of diversity to their underlying cause(s), but in the end, this is still just an educated guess. Our methodology, as described above, is based on the best practices described in Yaari and Kleinstein (20): stringent pre-processing, correcting for allelic differences between subjects, identification of insertions and deletions, and multiple clonal assignment methods for different datasets. ImmuneDB also provides the option of varying the stringencies of both data filtering and clonal assignment to ensure reproducible and robust results.

As a final argument for the efficacy of ImmuneDB, we show that repertoires analyzed with ImmuneDB take a form similar to those observed with other tools. In this section we compare ImmuneDB to a commonly used pipeline, MiXCR (6), on two datasets. First, we compare the germline gene assignment and clonal inference of the two methods on five samples, one each from five different tissues, all from one human organ donor. Second, we inspect how similar the overall view of a larger repertoire (19 biological replicates from a single organ donor's colon) appears with each method (1).

### Germline assignment and clonal inference

To determine how similarly MiXCR and ImmuneDB assign germline genes and infer clones, both pipelines were run on five samples from one human subject selected from Meng et al. (1) as listed in Table 2. The associated SRA accession information can be found in Table S1. This data set has a total of 651,988 reads. Sequences which were considered incorrect or misleading were discarded from both result sets: sequences had to have at least 160 bases in the V gene (at least all of CDR2), between 3 and 96 nucleotides in the CDR3, a functional V-gene assignment (no pseudogenes), and all V-gene calls (V-ties) for a given sequence had to be from the same V-gene family. For clonal comparisons, clones with only one total copy were discarded.

**Table 2:** Input reads for germline assignment comparison.

**Donor**	**Tissue**	**Input reads**
D181	Bone Marrow	185,057
D181	Ileum	140,084
D181	Jejunum	117,641
D181	Lung	113,325
D181	Peripheral Blood	95,881
	Total Reads	651,988

*Total number of input reads for germline assignment comparison between ImmuneDB and MiXCR. The total number of input reads was after pre-processing with pRESTO. The samples were selected from one of the deeply sequenced donors in Meng et al. (1)*.

#### Germline assignment

First, we compared which sequences each method was able to identify given this filtering. MiXCR identified 599,930 while ImmuneDB identified 611,252, and both identified the same 577,750. The corresponding Jaccard index of 0.91 indicates that the two methods identified a similar set of sequences.

Next, we compared how many of the identified sequences were assigned to the same genes. Since both methods allowed multiple assignments for both V genes and J genes, we considered two sequences to have the same gene call if the intersection of their gene calls contained at least one shared gene. For V genes, the two methods agreed on 98% of the sequences, for J-genes 95%, and when considering both genes, 93%. Of the sequences that differed with either gene, less than 1% differed in their gene family calls. Thus, overall both methods generally agreed on which germline genes gave rise to each sequence.

#### Clonal inference

Next, we compared how similarly the two methods inferred clonotypes for the 19 biologically independent colon sample replicates. The associated SRA accession information can be found in Table S2. For this process, we assigned each clone one or more labels from each method:

- If clone *A* from one method contained exactly the same sequences as clone *B* from the other, we labeled both “*identical*”.
- If clone *A* from one method contained a strict superset of sequences compared to clone *B* from the other method, we labeled *A* “*superset*” and *B* “*subset*”.
- If clone *A* from one method contained a portion of sequences compared to a clone *B* from the other method, we labeled both “*intersecting*”.
- If clone *A* from one method was disjoint from all clones from the other method, we labeled it “*disjoint*”.

Note that a clone could potentially have both the labels *superset* and *intersecting* simultaneously if it contained all the sequences from a clone inferred by the other method and contained sequences from another clone. Further, a clone could have multiple *superset* labels if it contained all the sequences from multiple clones inferred from the other method.

As shown in Table 3, ImmuneDB inferred 13,736 clones whereas MiXCR inferred 14,453. Of these 10,786 were identical; that is both methods constructed clones with exactly the same set of sequences. In 1,665 cases, an ImmuneDB clone was a subset of a MiXCR clone. There are two reasons this occurred. First, different amounts of *N* nucleotides in the either the V- or J-region can cause sequences, that are otherwise similar, to be assigned different sets of gene ties and therefore placed in different clones. Second, since ImmuneDB requires *pairwise* 85% similarity of CDR3 amino-acid sequences in clones, some sequences that may actually originate from the same clone are separated. Conversely, 2,819 MiXCR clones are subsets of an ImmuneDB clone. Nearly all of these are due to overly-strict J-gene assignment, resulting in separation of likely clonally related sequences. For example, some sequences that are one nucleotide away from IGHJ1 and two away from IGHJ4 could easily be confused due to sequencing error (4).

**Table 3:** Label assignments for clonal inference comparison.

**Clone Type**	**ImmuneDB**	**MiXCR**
Identical	10,788	10,788
Subset	1,665	2,819
Superset	1,155	726
Intersecting	22	12
Superset & Intersecting	72	74
Disjoint	36	36

*Clonal labels when comparing methods of clonal inference between ImmuneDB and MiXCR. Identical indicates the same set of clones was identified by both methods, subset/superset means the clone constructed by the associated pipeline was a subset/superset of one assigned by the other pipeline, and intersecting means there some sequences from a clone assigned by one pipeline that overlapped sequences assigned by the other pipeline*.

### Overall repertoire features

Repertoire analysis pipelines should reveal similar overall trends in acceptably large datasets even if the minutiae of sequence assignment and clonal inference differ. Specifically, when looking at sufficiently large clones, the overlap across samples and diversity should lead to similar conclusions. It is generally acceptable to only look at larger clones as smaller clones have likely been under-sampled or are an artifact of sequencing error (21).

To compare repertoire-level metrics generated from ImmuneDB and MiXCR processed data, 19 biologically independent colon replicates were analyzed. We assessed the similarity of the two pipelines by comparing their clone size distributions, diversity measures, rarefaction, and clonal overlap between samples as described in Meng et al. (1).

#### Clone size distribution

**Figure 4:**
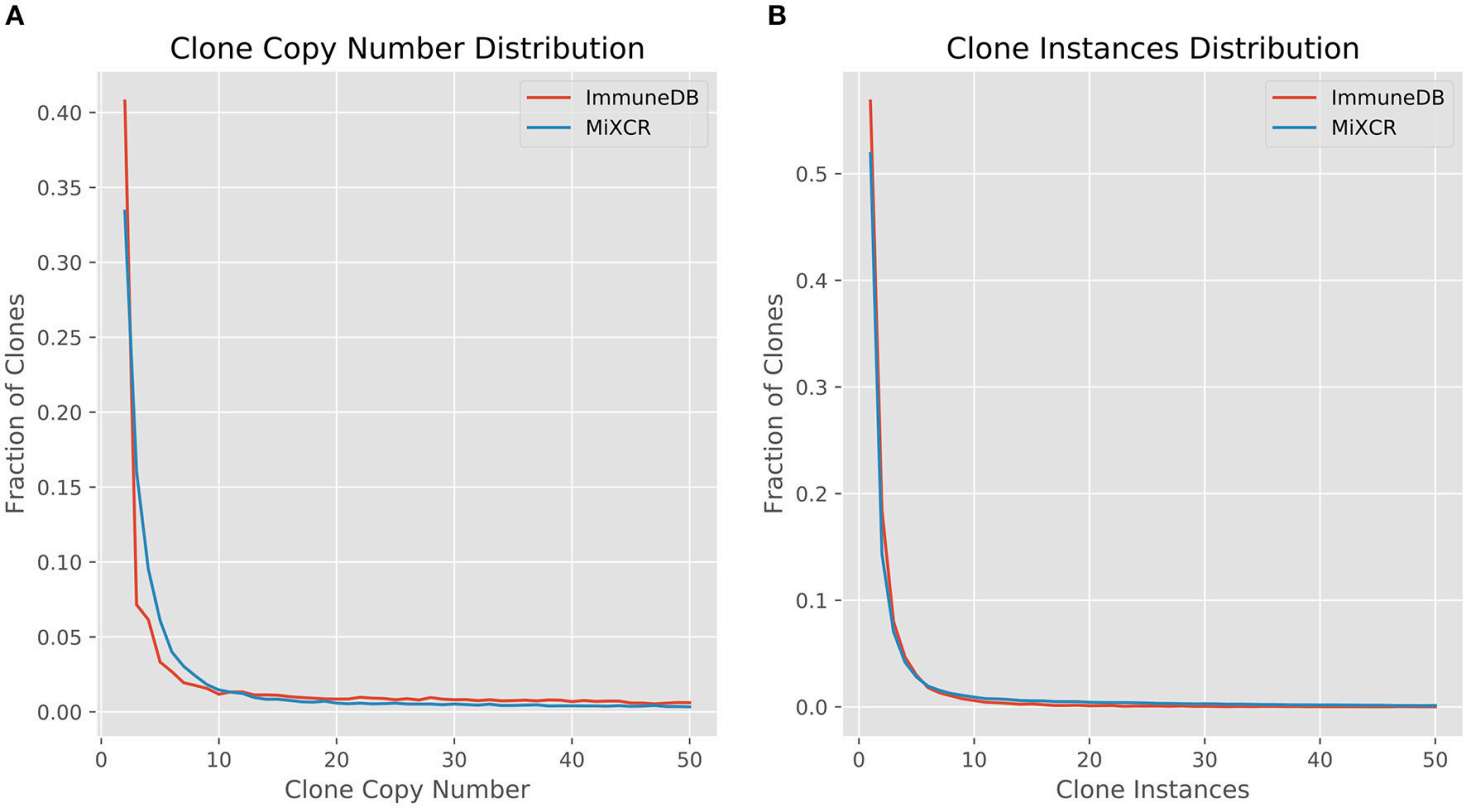
Comparison of clone size distributions between ImmuneDB and MiXCR in 19 colon samples subjected to bulk antibody heavy chain V-region sequencing from one organ donor [data from (1)]. Clone size is given as copies in **(A)** and instances in **(B)**. Both plots have been restricted to a maximum X-value of 50, but the trends continue beyond that.

We first looked at clone size distributions from the two pipelines. In Figure 4, the left panel shows a comparison of clone sizes as measured by copy number. The overall landscape is similar with both methods, especially when looking only at clones with 10 or more sequence copies. For smaller clones, the difference in clone sizes can be attributed to the more stringent CDR3 similarity measure MiXCR uses compared to ImmuneDB. The right panel shows the same comparison but instead measures the size of clones as the number of instances comprising the clone. Both methods have nearly identical clone size distributions, especially when considering clones with at least 2 instances.

#### Diversity

We next considered the diversity of the clones assigned by each method. The diversity index ^*q*^*D*, as defined by Equation 1 quantifies how many different clones there are.

Equation 1: Diversity index ^*q*^*D*

$$q_{D}={\left(\overset{R}{\underset{i\;=1}{\sum^{\text{​}}}}p_{i}^{q}\right)}^{1/(1-q)}$$

**Figure 5:**
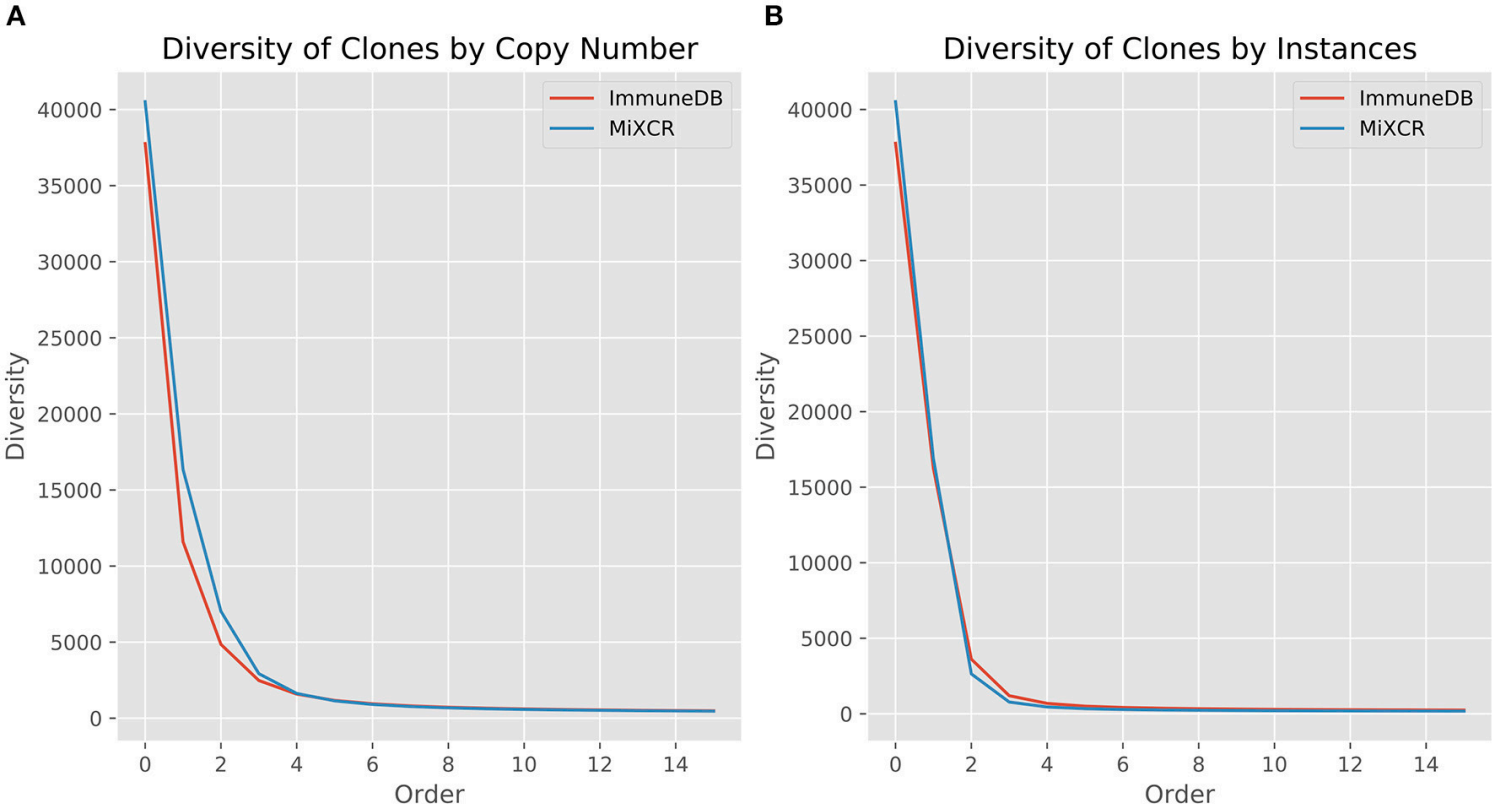
Comparison of diversity calculations between ImmuneDB and MiXCR in 19 colon samples from one donor (same data as in Figure 4). As order increases, more weight is given to larger clones. For both copies and instances, MiXCR inferred more diverse clonal populations for low order numbers. As order increases, however, the two methods begin to converge.

Here, *R* is the number of clones (richness), *p*_*i*_ is the fraction of the repertoire (either as copies or instances) inferred to be in clone *i*, and *q* is the order. When the order is zero, the diversity is richness, or total number of clones. Increasing the order, *q*, gives more weight to the larger clones (21, 22). Figure 5 shows the diversity at orders 1 through 15 for ImmuneDB and MiXCR, measuring clone size both as copies and instances.

It is clear that MiXCR infers more clones than ImmuneDB. However, when the order number is increased (more weight is given to large clones) the diversity of the two methods converges.

#### Rarefaction

**Figure 6:**
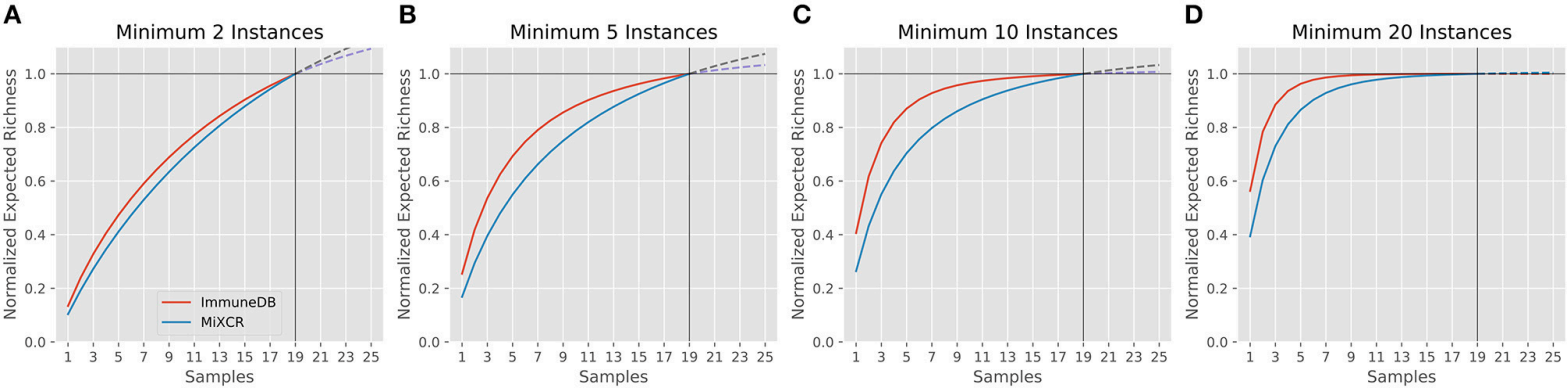
Rarefaction analysis for both ImmuneDB and MiXCR for clone size cutoffs of 2 instances **(A)**, 5 instances **(B)**, 10 instances **(C)**, and 20 instances **(D)** in 19 colon samples from one donor. The Y-axis shows the number of predicted clones when the population has been sampled between 1 and 25 times. A rarefaction curve that plateaus indicates the underlying clonal population has been adequately sampled. For all cutoffs, although the overall richness varies, the conclusion drawn would likely be the same: for clones under 10 instances, more sampling is required, while larger clones have been sampled sufficiently.

Rarefaction gives insight into how many clones are estimated to occur given a certain number of samples (biological replicates) from the same source. A rarefaction curve that levels out indicates that fewer new clones will be found with further sampling. Figure 6 shows the rarefaction curves for ImmuneDB and MiXCR for clones with at least 2, 5, 10, and 20 instances. The x-axis shows the number of samples and the y-axis shows the normalized richness (the richness divided by the richness at 19 samples). The solid lines (up to 19) are calculated from the 19 samples being compared, whereas the dashed lines past sample 19 show the projected number of additional clones if more replicates had been acquired. As only larger clones are considered, the rarefaction curves both begin to level out, indicating that those larger-clone populations have been more adequately sampled. Both pipelines tended to agree on when clones had been sampled enough, even though the overall diversity appears to be higher with MiXCR (indicated by lower fractional richness).

#### Sample overlap

We next evaluated the amount of clonal overlap using the cosine similarity, as defined by Equation (2):

Equation 2: Cosine similarity between vectors *A* and *B*

$$C\left(A,B\right)=\frac{\sum_{i=1}^{n}A_{i}B_{i}}{\sqrt{\sum_{i=1}^{n}A_{i}^{2}}\sqrt{\sum_{i=1}^{n}B_{i}^{2}}}$$

In this case, *A* and *B* are vectors corresponding to two samples both of which have a length equal to the total number of clones in the dataset. The *i*th value in each vector indicates the number of copies of the *i*th clone in the sample represented by the vector.

**Figure 7:**
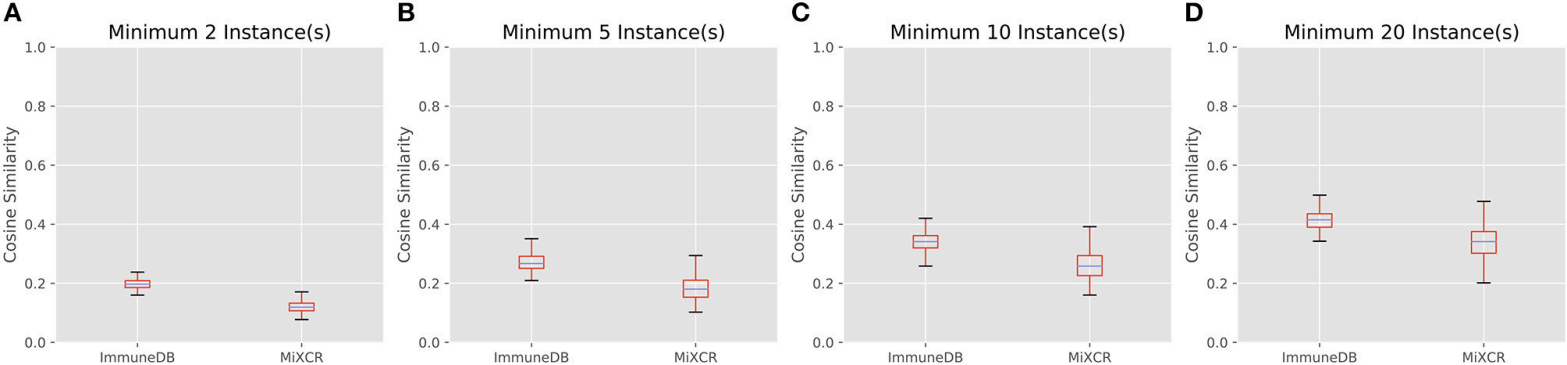
The distribution of cosine similarities (a measure of clonal overlap) between each pair of 19 colon samples for clone size cutoffs of 2 instances **(A)**, 5 instances **(B)**, 10 instances **(C)**, and 20 instances **(D)**. The boxes represent the interquartile range, the purple lines are the median, and the whiskers show the range of data. MiXCR finds less overlap across samples, because it has a more stringent CDR3 similarity requirement for clonal sequences than ImmuneDB, but both methods show a similar trend: larger clones overlap across more samples as one may expect.

Figure 7 shows the cosine similarity for clones with a minimum of 2, 5, 10, and 20 sequence instances. MiXCR infers less overlap between samples, but the general trend between both methods is the same: as expected, with larger clones, more overlap is discovered. Further, the distribution of cosine similarities about the median of each method are not significantly different. That is for both methods, clones over a given instance count tend to be distributed across a similar number of samples with a similar fraction of sequences in each sample.

## Discussion

ImmuneDB provides a unified method for the storage and analysis of large amounts of high-throughput immune receptor sequencing data. Like other pipelines such as Change-O (5) and MiXCR (6), it can analyze data from raw reads through clonal assignment. ImmuneDB has two method of germline calculation, anchoring and local-alignment, and provides the option of filtering the data at different QC and copy number cut-offs, which is desirable when samples with different sequencing depths are being compared. In addition, ImmuneDB provides multiple methods of clonal assignment. Combined, these features provide a variety of ways to analyze different types of data.

ImmuneDB is also flexible in that it can import pre-annotated data in a variety of formats supported by other AIRR software tools. This allows users to use custom tools for their dataset, using ImmuneDB for only a portion of the analysis. To provide a comprehensive suite of repertoire analysis tools, ImmuneDB also integrates downstream analyses such as selection pressure via BASELINe (9), lineages via clearcut (13), and novel allele detection via TIgGER (11), reducing the need for users to learn individual tools. Unlike most other tools, ImmuneDB stores the data in an easily queryable MySQL database and provides a web-interface for easily sharing data with non-technical users.

It is worth noting that ImmuneDB does make some assumptions when using other tools, however. For example, it is assumed that sequences in a clone have the same V gene, J gene, and CDR3 length and that they come from the same organism. Although generally this is likely acceptable, there are certain situations where such assumptions may not hold, such as donor/recipient data where a clone may span multiple recipients. As such, it is important to consider the limitations of all tools before using them on non-traditional datasets.

Additionally, since ImmuneDB calculates clones on a per-subject basis, adding new samples to a subject requires clonal inference to be re-run for that subject. However, the rest of the database will remain unchanged.

Finally, in section Comparison to MiXCR we compared ImmuneDB to MiXCR, a pipeline that similarly determines germline usage and infers clonotypes to show that the benefits of using ImmuneDB do not come at the cost of drastically changing conclusions one may draw from the data. Although the methods differ in their approach to clonal assignment, both yield similar clone size distributions, rarefaction plateau points, and sample overlap.

## Conclusion and future plans

In this paper we have provided a comprehensive description of ImmuneDB, a system for the analysis of large-scale, high-throughput immune repertoire sequencing data. ImmuneDB can operate either independently, providing an integrated collection of analysis tools to process raw reads for gene usage, infer clones, aggregate data, and run downstream analyses, or in conjunction with other AIRR tools using its import and export features. Thus, ImmuneDB can be an all-in-one solution for repertoire analysis or serve as an efficient way to visualize and store annotated repertoire data, or both. In either case, the ImmuneDB web-interface can be used to easily interact with the underlying dataset.

ImmuneDB is regularly being updated to address user needs and handle the increasing complexity of adaptive immune receptor repertoire sequencing data. In the future we plan to add a feature to allow users to assess the quality of individual sequencing libraries (replicates) before running the entire pipeline. As pRESTO provides a per-sequence quality control step, this new feature will provide a post-identification quality control step, informing users if their samples have insufficient depth or quality. Further, the CDR3 similarity clonal inference method will receive two additional features. First, it will be extended to allow for different similarity thresholds for different CDR3 lengths. Second, this method will allow users to set a required minimum number of shared V-gene somatic hypermutations for sequences to be grouped into a clone.

## Author contributions

AR developed ImmuneDB and wrote the first draft of this manuscript. WM ran the sequencing experiments generating the data for this manuscript and helped test ImmuneDB. WM, EL, and UH provided input on important features to include in ImmuneDB and in this manuscript. EL and UH both contributed to editing this manuscript.

### Conflict of interest statement

The authors declare that the research was conducted in the absence of any commercial or financial relationships that could be construed as a potential conflict of interest.

## Footnotes

^1^https://github.com/arosenfeld/immunedb-frontend

^2^https://hub.docker.com/r/arosenfeld/immunedb/

^3^https://www.gnu.org/licenses/gpl-3.0.en.html

^4^http://www.imgt.org/IMGTScientificChart/Numbering/IMGTIGVLsuperfamily.html

^5^https://immcantation.readthedocs.io

## Supplementary material

Table S1 SRA accession information for the five samples used in the germline assignment comparison between ImmuneDB and MiXCR.

Table S2 SRA accession information for the 19 samples used in the clonal inference comparison between ImmuneDB and MiXCR.
